# Zebrafish as a model organism for virus disease research: Current status and future directions

**DOI:** 10.1016/j.heliyon.2024.e33865

**Published:** 2024-07-02

**Authors:** Sofyantoro Fajar, Sendi Priyono Dwi, Indah Septriani Nur, Aristyaning Putri Wahyu, Mamada Sukamto S, Adipuri Ramadaningrum Winda, Wijayanti Nastiti, Frediansyah Andri, Nainu Firzan

**Affiliations:** a Faculty of Biology, Universitas Gadjah Mada, Yogyakarta, 55281, Indonesia; b Department of Pharmacy, Faculty of Pharmacy, Hasanuddin University, Makassar, 90245, Indonesia; c National Agency of Drug and Food Control (BPOM), Jakarta, 10560, Indonesia; d Research Center for Food Technology and Processing (PRTPP), National Research and Innovation Agency (BRIN), Yogyakarta 55861, Indonesia

## Abstract

Zebrafish (*Danio rerio*) have emerged as valuable models for investigating viral infections, providing insights into viral pathogenesis, host responses, and potential therapeutic interventions. This review offers a comprehensive synthesis of research on viral infections using zebrafish models, focusing on the molecular mechanisms of viral action and host-virus interactions. Zebrafish models have been instrumental in elucidating the replication dynamics, tissue tropism, and immune evasion strategies of various viruses, including Chikungunya virus, Dengue virus, Herpes Simplex Virus type 1, and Influenza A virus. Additionally, studies utilizing zebrafish have evaluated the efficacy of antiviral compounds and natural agents against emerging viruses such as SARS-CoV-2, Zika virus, and Dengue virus. The optical transparency and genetic tractability of zebrafish embryos enable real-time visualization of viral infections, facilitating the study of viral spread and immune responses. Despite challenges such as temperature compatibility and differences in host receptors, zebrafish models offer unique advantages, including cost-effectiveness, high-throughput screening capabilities, and conservation of key immune pathways. Importantly, zebrafish models complement existing animal models, providing a platform for rapid evaluation of potential therapeutics and a deeper understanding of viral pathogenesis. This review underscores the significance of zebrafish research in advancing our understanding of viral diseases and highlights future research directions to combat infectious diseases effectively.

## 1 Introduction

The ongoing threat of infectious diseases caused by viral pathogens remains a formidable challenge to global public health [1,2]. Viruses have the ability to invade host cells, hijack their cellular machinery, and manipulate their molecular processes, leading to a cascade of pathological events [3,4]. Consequently, comprehending the intricate mechanisms underlying viral pathogenesis is crucial for devising effective therapeutic strategies to combat these diseases. Viral pathogenesis encompasses a complex interplay between the invading virus and the host immune system, as well as the specific tissue tropism and cellular targets of the virus [5,6]. Viruses employ various strategies to evade or subvert the immune response, allowing them to establish infection and propagate within the host [7,8]. Understanding these strategies and the molecular interactions involved is pivotal in unraveling the intricate dance between the virus and the host immune defenses. Moreover, deciphering the molecular basis of viral replication and the key viral proteins involved in this process is essential for identifying potential targets for therapeutic intervention. Viral replication often relies on the exploitation of host cellular machinery, including enzymes, receptors, and signaling pathways [[4], [5], [6]]. By elucidating the intricate molecular interactions between the virus and host factors, researchers can identify critical vulnerabilities that can be targeted for the development of antiviral therapies. In addition to understanding viral pathogenesis, the development of effective therapeutic strategies is imperative to mitigate the impact of viral diseases. Antiviral therapies aim to impede viral replication, inhibit viral entry into host cells, or modulate the host immune response to enhance viral clearance [9]. Various approaches have been pursued, including the development of small molecule inhibitors, monoclonal antibodies, identification of microbial natural products, and nucleic acid-based therapeutics [[10], [11], [12], [13]]. Additionally, the repurposing of existing drugs that exhibit antiviral activity against specific viral targets has shown promise in rapidly identifying potential treatments [14,15].

In order to advance our understanding of human viral infections and unravel the intricate molecular and cellular processes involved, it is of utmost importance to employ research model organisms that are both reliable and versatile [[16], [17], [18]]. Reliable research models should accurately mimic the course of the disease, encompassing the progression from viral entry and replication to the manifestation of symptoms and immune responses. Versatility is another crucial attribute of research models in the study of human viral infections. The models should possess the capacity to accommodate a wide range of experimental designs and allow for the exploration of various facets of viral infections. This includes the evaluation of different viral strains or variants, testing the efficacy of antiviral compounds or vaccines, and deciphering the mechanisms of viral transmission and pathogenesis. One such model organism that has gained prominence in the field of viral pathogenesis research is the zebrafish (*Danio rerio*) [[19], [20], [21]]. Zebrafish offers several advantages as a research model organism for studying viral infections (Table 1). Zebrafish have a well-characterized immune system that shares remarkable similarities with humans, including the presence of immune cell types and conserved immune pathways [20,22]. This similarity allows researchers to investigate the host immune responses to viral infections and evaluate the efficacy of antiviral therapies in a biologically relevant context. Also, zebrafish are optically transparent during early development, enabling real-time visualization of viral infection dynamics and host responses at the whole-organism level [23]. Additionally, the implementation of Europe-wide legislation, exemplified by the EU Directive 2010/63/EU, represents a significant step toward standardizing the use of zebrafish in research [24]. This directive emphasizes the onset of independently feeding larval development as a crucial criterion, indicating when experiments necessitate regulatory approval due to the assumed presence of pain and distress. As a result, until larvae reach the stage of independent feeding, typically up to 5 days post-fertilization, they are not classified as experimental animals, thereby exempting them from the need for ethics committee approval [24].

**Table 1:** Advantages of zebrafish as a model organism for studying human viral infections.

Advantage	Description	Example
Well-characterized immune system	Zebrafish show immune responses like mammals, including both innate and adaptive immunity. This model aids in studying host-virus interactions and inflammation, providing insights into human viral infections.	Sullivan et al. used a zebrafish model to understand host immune cell reactions after being infected to human IAV [25].
Optical transparency during early development	The transparency of zebrafish embryos enables clear observation of internal organs and tissues simplifying research on various conditions, including viral diseases.	CHIKV and SINV cause viral encephalopathies, but their entry into the central nervous system (CNS) is still unclear. Research in zebrafish showed these viruses quickly inhabited brain tissue and persist there. CHIKV targets blood-brain barrier endothelial cells, while SINV infects peripheral nerve endings and spreads via axonal transport [26,27].
Fluorescent reporter viruses and genetically modified zebrafish lines	Gene knockdown and knockout methods on zebrafish are used to understand gene effects on viral infection. Screening mutated zebrafish against various viruses could also provide key insights into viral diseases at the molecular level.	It was revealed that by editing the FTR42 genome, zebrafish experienced an improvement of survival against the spring viremia of carp virus facilitated by their ability to enhance IFN immunity [28].
Drug screening capabilities	Zebrafish models of human viral diseases offer the chance to study the interaction between viruses and hosts and perform genetic and chemical screenings. These models assist in developing and testing novel antiviral approaches.	Sullivan et al. employed zebrafish models of human IAV infections to screen antiviral medications [25]. Antoine et al. showed that zebrafish is a great model for screening of *anti*-HSV compounds [29].

Abbreviations: CHIKV, chikungunya virus; HSV, herpes simplex virus; IAV, Influenza A virus; SINV, Sindbis viruses.

The availability of fluorescent reporter viruses and genetically modified zebrafish lines expressing fluorescent proteins in specific cell types or tissues has facilitated the study of viral tropism, replication, and spread [21]. Moreover, the zebrafish model has also demonstrated its utility in drug screening and evaluation of potential antiviral compounds [30]. Zebrafish larvae can be exposed to viral infections and treated with small molecules or compounds to assess their efficacy in inhibiting viral replication or mitigating viral-induced pathologies. High-throughput screening approaches using zebrafish have identified novel antiviral compounds with potential therapeutic applications against viral infections [[31], [32], [33]].

In this review, we aim to provide a comprehensive overview of the applications of zebrafish as a model organism in studying the pathogenesis of human viral infections. We focus on specific viruses and highlight key findings, methodologies, and therapeutic approaches that have been explored using zebrafish models. By synthesizing the available literature, this review underscores the value of zebrafish in advancing our understanding of viral pathogenesis and in identifying potential targets for therapeutic intervention. Several studies have successfully utilized zebrafish to investigate the pathogenesis of various viral infections. For instance, researchers have employed zebrafish models to study the replication, tissue tropism, and transmission routes of viruses such as Chikungunya virus (CHIKV) [[26], [34], [35]], Sindbis virus (SINV) [27,36], Dengue virus (DENV) [37], Human Noroviruses (HuNoV) [[38], [39], [40], [41]], Herpes Simplex Virus type 1 (HSV-1) [[29], [42], [43], [44]], Hepatitis C virus (HCV) [[45], [46], [47]], Influenza A virus (IAV) [[25], [48], [49]], Zika virus (ZIKV) [50,51], Severe acute respiratory syndrome coronavirus 2 (SARS-CoV-2) [[52], [53], [54], [55], [56], [57], [58], [59], [60], [61], [62]], and Human cytomegalovirus (HCMV) [63]. These studies have provided valuable insights into viral entry mechanisms, viral replication kinetics, host immune responses, and the development of antiviral therapeutic strategies.

## 2 Understanding human viral pathogenesis through zebrafish models

**Fig. 1:**
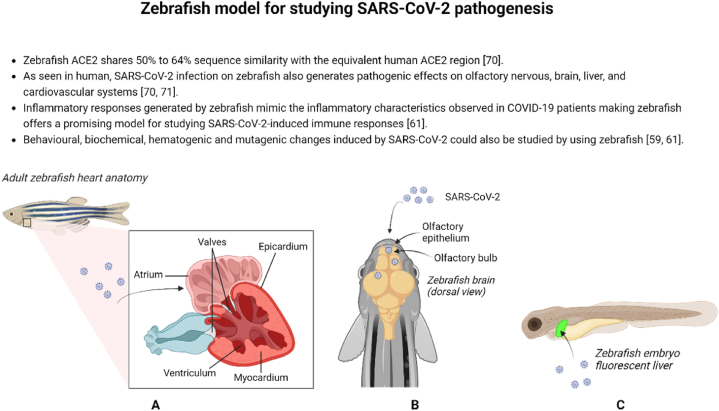
Zebrafish models for studying SARS-CoV-2 pathogenesis. Zebrafish have become an important model organism for investigating the effects of SARS-CoV-2 viral proteins and related pathologies. Numerous studies have examined how specific segments of the viral protein affect various organ systems in zebrafish, including the cardiovascular (A), olfactory (B), and hepatic systems (C) providing insights into the mechanisms behind SARS-CoV-2-induced damage.

**Fig. 2:**
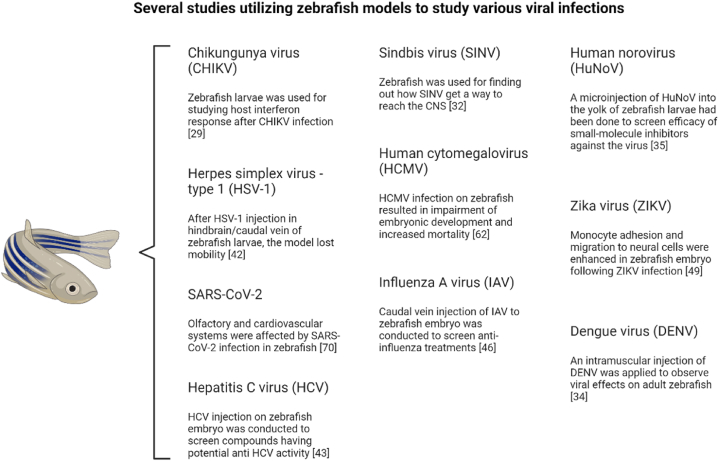
Utilizing zebrafish models to study various viral infections. Researchers have used zebrafish models to study the replication, tissue tropism, and transmission routes of various viruses. These studies have yielded valuable insights into viral entry mechanisms, replication kinetics, host immune responses, and the development of antiviral therapies.

Zebrafish have been used as a model for deciphering the pathogenesis of several viruses infecting humans (Fig. 1, Fig. 2) as described below.

### 2.1 Severe acute respiratory syndrome coronavirus 2 (SARS-cov-2)

SARS-CoV-2, classified taxonomically as a member of the Betacoronavirus genus and Coronavirinae family, causes the pandemic of Coronavirus disease 2019 (COVID-19) [64,65]. SARS-CoV-2 possesses a single-stranded, positive-sense RNA genome with specific structural proteins, including spike (S), membrane (M), envelope (E), and nucleocapsid (N) proteins [66]. SARS-CoV-2 shares genetic similarities with other coronaviruses such as SARS-CoV and MERS-CoV [67]. The virus causes a range of symptoms, from mild to severe illness, including respiratory, musculoskeletal, neurological, cardiovascular, renal, and digestive symptoms [[68], [69], [70], [71], [72]].

In the quest to develop suitable models for studying SARS-CoV-2 infection, researchers have explored the use of zebrafish. Extensive experiments to infect zebrafish larvae with SARS-CoV-2 have been reported [52]. The study found that bath exposure to the virus did not lead to successful infection, as viral RNA was undetectable after two days. Microinjection of the virus into different sites of the larvae revealed that injection into the yolk did not result in active viral replication, while injection into the swim bladder showed evidence of abortive infection in a subset of larvae. However, the amount of infectious particles produced was too small to be detected. The swim bladder, which is homologous to the human lung, appeared to be the most permissive organ for infection [52]. The lack of infectivity in zebrafish larvae could be attributed to the absence of a suitable receptor or intrinsic immune mechanism. Overexpression of human Angiotensin-converting enzyme 2 (ACE2), the receptor for SARS-CoV-2, did not result in infectivity in zebrafish larvae or fish cell lines. Therefore, Laghi et al. (2022) suggested further optimization of infection protocols, including the development of transgenic zebrafish expressing human ACE2.

To address this limitation, using an alternative methodology, a humanized zebrafish model was constructed by transplanting human alveolar epithelial cells (A549) into the swim bladder of adult zebrafish [54,73]. The anatomy and cytology of the swim bladder were examined after seven days, confirming successful xeno-transplantation and adherence of human respiratory epithelial cells. Following confirmation, fish were injected with SARS-CoV-2 spike protein to induce pathological features. Key pathologies induced by the spike protein included increased mortality, behavioral fever, renal cell necrosis, skin hemorrhages, and swim bladder inflammation. Based on these results, the researchers proposed that this xenotransplant model of humanized zebrafish may help to overcome the limitations of the zebrafish model for studying COVID-19. In a separate study, Choi et al. (2022) investigated the mechanism of host cell entry of SARS-CoV-2 using a pseudotyped lentivirus in zebrafish embryos/larvae as an in vivo model [55]. The successful entry of the pseudovirus was confirmed by luciferase gene expression, which was inhibited by chloroquine and bafilomycin A1. Administering chloroquine, known for its broad-spectrum antiviral properties hindering membrane fusion, or bafilomycin A1, which specifically targets vacuolar proton ATPases to impede endolysosomal trafficking, notably decreased luciferase expression in this study, suggesting the potential role of the endolysosomal system in the viral entry process. Importantly, Choi et al. (2022) demonstrated the expression of ACE2 and two-pore channel subtype 2 (TPC2) in the peripheral sense organs of zebrafish embryos/larvae, indicating their potential role in viral entry. These findings highlight zebrafish embryos/larvae as a suitable model for studying SARS-CoV-2 host cell entry. Taken together, these studies highlight the potential of zebrafish as a valuable model for investigating SARS-CoV-2 infection and host-cell interactions. The humanized zebrafish model provides a platform to study COVID-19 pathology and test potential therapeutics, while zebrafish embryos and larvae offer insights into the mechanisms of viral entry. Further optimization and development of these models could contribute to our understanding of the virus and aid in the development of effective interventions.

Zebrafish have emerged as a valuable model organism for studying the effects of SARS-CoV-2 viral proteins and understanding the associated pathologies. Several studies have explored the impact of specific portions of the viral protein on different organ systems (e.g., cardiovascular (Fig. 1A), olfactory (Fig. 1B), and hepatic systems (Fig. 1C)) in zebrafish, shedding light on the mechanisms underlying SARS-CoV-2-induced damage. Intranasal delivery of SARS-CoV-2 S receptor binding domain (RBD) in adult zebrafish results in severe olfactory damage characterized by cilia loss, edema, hemorrhages, and necrosis [56]. The study demonstrates that the damage primarily affects non-sensory regions of the olfactory lamellae, leading to indirect impairment of olfactory sensory neurons (OSNs) and severe olfactory dysfunction. However, interestingly, functional recovery occurs relatively quickly compared to mammalian models. The loss of olfaction is observed in response to both food and bile odorants, indicating dysregulation of ciliated and microvillous OSNs. Kraus et al. (2022) also showed that the zebrafish olfactory organ (OO) shows dynamic changes, including proliferation and apoptosis of certain cell types in response to SARS-CoV-2 S RBD damage. However, the exact mechanism of SARS-CoV-2 S RBD-induced olfactory damage remains unclear. In a 2022 study by Ventura Fernandes et al. (2022), zebrafish injected with a specific portion of the SARS-CoV-2 spike protein (rSpike) exhibited adverse effects on the liver, nervous system, and reproductive system [57]. Histological analysis revealed liver abnormalities, including lymphocyte infiltration, sinusoidal dilation, necrosis, and steatosis. Female zebrafish experienced severe damage to the ovary, but the damage was partially reversed after a second injection of rSpike. The brain also showed an inflammatory response without histopathological lesions. The study also demonstrates that the high toxicity of the spike protein in zebrafish suggests its potential for assessing vaccine responses and exploring therapeutic strategies.

Tyrkalska et al. (2022) revealed that zebrafish larvae, unlike human and mouse macrophages, mount a swift innate immune reaction to various forms of the SARS-CoV-2 spike protein, including the full-length protein [58]. Therefore, this study demonstrates that the use of zebrafish as an animal model addresses the limitations observed in human and mouse macrophages, offering valuable insights into COVID-19-associated cytokine storm syndrome (CSS). The spike protein injected into the hindbrain of zebrafish larvae induced the expression of proinflammatory genes, activation of NFκB (Nuclear factor kappa B), and the recruitment of neutrophils and monocytes. Tyrkalska et al. (2022) also showed that different variants of the spike protein exhibited distinct proinflammatory properties. Taken together, this study demonstrates that zebrafish hold promise for screening anti-inflammatory compounds targeting COVID-19-associated CSS. In a subsequent investigation, the zebrafish model was employed to examine the impact of S1WT (S1 domain of Spike protein from the Wuhan strain) of SARS-CoV-2 [74]. The results demonstrate that S1WT induces hyperinflammation in zebrafish larvae via the Tlr2/Myd88 (Toll-like receptor 2/Myeloid differentiation primary response 88) signaling pathway. Moreover, Cai et al. (2023) utilized transgenic zebrafish to evaluate the cytotoxicity of individual SARS-CoV-2 viral proteins on renal tubular epithelial cells [59]. By expressing viral proteins in a renal tubular cell-specific manner, they observed that zebrafish expressing the ORF3A (Open Reading Frame 3 A) protein displayed a significant pericardiac effusion phenotype, resembling other zebrafish models of acute kidney injury. These findings provide evidence for the cytotoxic effects of specific SARS-CoV-2 viral proteins on renal tubular cells, highlighting the potential mechanisms underlying renal injury in COVID-19. These findings offer valuable insights into the understanding of how fish respond to viral proteins at the host level. Collectively, these studies underscore the value of zebrafish as a model organism for investigating the effects of SARS-CoV-2 viral proteins on various organ systems and provide important insights into the pathogenesis of COVID-19.

A series of studies have shed light on the ecotoxicity and physiological impacts of SARS-CoV-2 on zebrafish. A study by Luz et al. (2023) provides novel insights into the ecotoxicity of SARS-CoV-2, demonstrating that exposure to the virus induces changes in habituation memory behavior, antipredatory response in shoals, and mutagenic effects in zebrafish [60]. Researchers in this study observed alterations in the habituation memory of the animals, suggesting behavioral impairment. Furthermore, zebrafish exposed to SARS-CoV-2 showed a diminished response to the predatory stimulus, indicating a negative impact on antipredatory behavior. Additionally, exposure to SARS-CoV-2 resulted in biochemical changes indicative of redox imbalance, cholinesterase inhibition, nitrosative stress, and an inflammatory response. The mutagenic effects of SARS-CoV-2 were evident, as evidenced by an increase in erythrocyte nuclear abnormalities and DNA damage. The observed alterations in behavior and biochemical parameters suggest disruptions in underlying neural networks and physiological mechanisms. Zheng et al. (2021) focused on the impact of the SARS-CoV-2 spike protein on blood coagulation using zebrafish embryos [61]. Microinjection of the spike protein resulted in reduced bleeding time and the formation of thrombi in blood vessels and capillaries. Confocal imaging revealed plaque formation in the wall of the dorsal aorta. Thrombosis occurred rapidly, within 3–5 min, indicating a direct effect of the spike protein on blood clotting. This study also showed that the spike protein competes with anticoagulation factors, such as antithrombin (AT) and heparin cofactor II (HCII), for binding to heparin, thereby inhibiting the inactivation of thrombin. This interference with the normal anticoagulation process may contribute to the excessive coagulation seen in severe COVID-19 cases, acting as an additional independent factor in the development of the inflammatory storm.

In a computational analysis presented by Bastos et al. (2023), the researchers predicted protein-protein interactions of SARS-CoV-2 Spike-derived peptides in humans and zebrafish [62]. Two peptides, PSPD2002 and PSPD2003, were chosen based on their antigenic potential. Zebrafish larvae were immunized with these peptides, and their effects were evaluated [62]. Larvae injected with PSPD2002 and PSPD2003 exhibited lower survival rates compared to the control group. Histopathological analysis revealed intense inflammatory infiltration in various organs of PSPD2003-injected fishes. The peptides also influenced the recruitment and phenotype of immune cells, particularly macrophages. PSPD2002 induced a faster inflammatory response, while PSPD2003 led to a prolonged and pro-inflammatory process. These findings mimic the inflammatory characteristics observed in COVID-19 patients. Therefore, the study may provide insights into the inflammatory process of the disease and contribute to understanding the pathophysiology of COVID-19. Collectively, these studies provide valuable insights into the ecotoxicity, physiological impacts, and inflammatory responses induced by SARS-CoV-2 in zebrafish. The research highlights the multifaceted effects of the virus on behavior, blood coagulation, and immune responses, facilitating a better understanding of the pathophysiology of COVID-19 and its potential ecological implications.

### 2.2 Chikungunya virus (CHIKV)

Chikungunya virus (CHIKV) is a member of the Alphavirus genus and is characterized by its possession of a single-stranded, positive-sense RNA genome [75]. The infection with CHIKV leads to acute febrile illnesses accompanied by severe joint pain, known as arthralgia [76]. These debilitating symptoms significantly impact affected individuals, resulting in a substantial burden of morbidity [77]. Moreover, CHIKV infection poses a significant public health concern due to its potential for widespread transmission and the subsequent impact on affected communities [78,79]. Although primarily transmitted by mosquitoes and prevalent in regions such as Africa, Asia, and the Indian subcontinent, CHIKV has demonstrated its ability to cause outbreaks in diverse regions, including the Americas [80,81].

To facilitate the investigation of CHIKV infection, researchers have successfully established a larval zebrafish model. This model has provided valuable insights into the infection process and its effects. Palha et al. (2013) utilized advanced imaging techniques to study CHIKV infection in zebrafish larvae, uncovering new knowledge about the dynamics of viral spread [26]. They observed that the pattern of viral spread in zebrafish larvae closely resembled that in mammals, with an initial peak of viremia at 24–48 h post-infection (hpi). Meanwhile, the expression levels of IFN-related genes such as *ifnϕ1* and *viperin* reached their peak at 17–24 hpi and maintained elevated levels for a minimum of four days. The appearance of newly infected cells occurred within 24 h of virus injection, indicating the initial set of infected cells. As the host interferon (IFN) response was activated, the appearance of newly infected cells decreased, suggesting increased resistance to the virus. Infected cells underwent apoptosis-mediated death, with the timing varying depending on the organ. Intriguingly, CHIKV persistence in the zebrafish brain suggested the potential of the brain as a reservoir for the virus, indicating that neurons might be an overlooked reservoir. This scenario is also applicable to infant humans, as encephalitis tends to occur in newborns with chikungunya disease rather than in adults. However, studies in adult macaques suggest that CHIKV persists in macrophages rather than in the central nervous system (CNS) [82]. Additionally, research in infected neonatal mice did not find evidence of CHIKV persistence in the brain [83]. Neutrophils and hepatocytes played a crucial role in producing IFN during CHIKV infection in zebrafish larvae, contrary to expectations. Neutrophils, in particular, constituted the majority of IFN-expressing leukocytes and demonstrated a significant role in controlling CHIKV infection. The unique advantage of the zebrafish model in this study lies in its ability to visualize infected cells and the production of IFN, facilitating the study of immune responses [26].

In addition to the insights gained through imaging techniques, microarray analysis has been employed to uncover the molecular mechanisms underlying CHIKV infection in larval zebrafish. Briolat et al. (2014) utilized microarrays to examine the gene expression profiles of CHIKV-infected zebrafish larvae [34]. The innate immune response to viral infection is primarily mediated by type I interferons (IFNs), which activate interferon-stimulated genes (ISGs) through JAK/STAT (Janus Kinase/Signal Transducers and Activators of Transcription) pathways [84,85]. By analyzing the transcriptional response of zebrafish larvae to CHIKV, Briolat et al. (2014) revealed a robust IFN response induced by CHIKV. They identified a core set of canonical ISGs conserved in both fish and mammals, along with lineage-specific gene families and species-specific noncoding RNAs. Moreover, the spatial expression pattern of ISGs showed tissue-restricted expression, particularly in the liver, vasculature, and gut.

In a follow-up study, Levraud et al. (2019) further explored the transcriptome of CHIKV-infected larvae using deep RNA sequencing, identifying specific genes induced by CHIKV infection independently of IFN [35]. These genes were associated with cytokine signaling, cytosolic DNA sensing, Toll-like receptor signaling, RIG-I-like receptor signaling, proteasome function, and herpes simplex infection. Collectively, these studies have provided significant insights into the host-virus interactions, viral replication dynamics, and host immune responses during CHIKV infection [34,35].

In summary, the larval zebrafish model has emerged as a valuable tool for studying CHIKV infection, enabling researchers to visualize viral spread, investigate the behavior of infected cells, and study immune responses. Advanced imaging techniques have shed light on the dynamics of viral spread, while microarray and transcriptome analysis have uncovered the molecular mechanisms underlying CHIKV infection and the role of IFN and ISGs. These findings contribute to a better understanding of CHIKV pathogenesis and the development of targeted interventions to combat CHIKV infection.

### 2.3 Sindbis virus (SINV)

Sindbis virus (SINV) is an enveloped RNA virus transmitted by mosquitoes, with occasional outbreaks reported in South Africa and Northern Europe. Human SINV infections are characterized by symptoms such as fever, rash, and persistent musculoskeletal pain resembling autoimmune diseases [86]. To gain insights into the infection course of SINV, researchers have turned to the zebrafish model as a valuable tool. Passoni et al. (2017) conducted a study using zebrafish larvae to investigate the progression of SINV infection [27]. By mimicking the natural entry route through intravenous injection, they observed that infected cells first appeared in the periphery before infecting neurons in the central nervous system (CNS). This observation suggested the presence of a host response in the periphery that limited the spread of the virus. Notably, the blood-brain barrier (BBB) remained intact, as there was no observed infection of endothelial cells or leakage into the brain parenchyma. Macrophages were found not to be the primary target of SINV, as depleting them did not prevent CNS infection. Instead, axonal transport was identified as an efficient mechanism for SINV spread within the brain. Passoni et al. (2017) successfully demonstrated that the use of fluorescent reporter viruses in the zebrafish model allowed for visualization of the infection progression and facilitated the study of neurotropic viruses.

Furthermore, the interplay between viral and bacterial pathogens in the context of SINV-infected larval zebrafish was explored by Boucontet et al. (2018). Their study focused on virus-induced bacterial hyper-susceptibility using SINV and *Shigella* as infecting agents [36]. The findings indicated that virus-induced defects in neutrophil function, such as compromised recruitment towards bacterial site infection and increased cell death of phagocytic neutrophil, contributed in elevating susceptibility to bacterial infection. Interestingly, this hyper-susceptibility was not limited to *Shigella* but could be applicable to other virus-bacterium combinations in zebrafish and humans. Therefore, these insights shed light on the intricate mechanisms involved in the pathogenesis of SINV and its potential consequences in the presence of co-infections [36].

Taken together, the zebrafish model has proven to be a valuable experimental system for studying the pathogenesis of SINV. By utilizing this model, researchers have gained insights into the progression of SINV infection, the role of axonal transport in viral spread within the brain, and the interplay between SINV and bacterial pathogens. These studies provide a better understanding of viral pathogenesis and highlight the complex nature of infectious diseases. Furthermore, the findings have the potential to contribute to the development of targeted therapeutic strategies aimed at mitigating the detrimental effects of SINV and its associated complications.

### 2.4 Dengue virus (DENV)

Dengue virus (DENV) is a causative agent of dengue, a viral hemorrhagic fever that poses a significant global public health threat, particularly in tropical and subtropical regions [87]. With millions of infections and illnesses occurring annually, DENV is primarily transmitted by mosquitoes and is characterized by four distinct serotypes, each capable of causing a range of disease manifestations [88]. Infection begins when DENV targets Langerhans cells and subsequently spreads to various hematopoietic cells, including macrophages and monocytes, leading to systemic dissemination of the virus [89].

To gain deeper insights into DENV infection dynamics and pathogenesis, researchers have successfully established an adult zebrafish model [37]. Utilizing this model, they replicated dengue viral pathology and investigated its clinical course. The study demonstrated that DENV infection in zebrafish led to notable changes in hematological parameters, including an increased white blood cell count, a decreased red blood cell count, and a decreased platelet count. Furthermore, the infected zebrafish exhibited liver damage and inflammation, providing valuable insights into the pathological consequences of dengue viral infection. The establishment of the adult zebrafish model for DENV research offers several advantages. It allows for the visualization and study of viral infection dynamics and host responses in real-time, providing a unique opportunity to unravel the intricate mechanisms underlying DENV pathogenesis. The ability to replicate key aspects of dengue viral pathology in the zebrafish model contributes to a better understanding of the disease and its clinical manifestations. Moreover, the model enables the evaluation of potential therapeutic interventions and the identification of novel targets for drug development.

### 2.5 Human Noroviruses (HuNoV)

Human noroviruses (HuNoV), members of the Caliciviridae family, are a major cause of gastroenteritis, resulting in significant morbidity, mortality, and economic burden [90]. Current models for studying HuNoV replication have limitations, including ethical concerns and short-lasting replication in mice [91,92]. Although some progress has been made with human B-cells and enteroids [93,94], there is a pressing need for simpler and more widely available HuNoV replication models.

To further our understanding of human norovirus infection, a larval zebrafish model has been developed and reported [38]. Van Dycke et al. (2019) found that multiple genotypes of HuNoV replicated efficiently in zebrafish larvae, showing a significant increase in viral genomes compared to other models such as mice and intestinal enteroids. Furthermore, the study demonstrates the successful passage of HuNoV from larva to larva. The study also showed that HuNoV replication was most prominent in the intestine of larvae one-day post-infection, with viral antigens also detected in hematopoietic tissue. Overall, this zebrafish larvae model represents a significant advancement in the understanding of HuNoV replication and provides a valuable tool for studying the biology of noroviruses and evaluating potential antiviral therapies.

Consistent with Van Dycke et al. (2019), Kim et al. (2022) found that the replication of HuNoV was prominently observed in zebrafish larvae, but not in adult zebrafish [39]. These findings emphasize the advantages of utilizing zebrafish larvae rather than adult zebrafish for HuNoV research. The larvae are more suitable for studying HuNoV due to their ability to support viral replication and provide consistent responses to infection. In contrast, adult zebrafish may possess a well-developed adaptive immune system or produce antibodies against HuNoV, which could hinder virus replication. Kim et al. (2022), additionally showed that genes linked to transmembrane transporters, aiding in balancing ion exchange in the intestine during infection, heat shock chaperones, a common cellular response to viral proteins, and cytokines, signifying the activation of adaptive immunity in response to virus exposure, were highly expressed in HuNoV-infected zebrafish. Therefore, this study successfully identified biomarkers associated with HuNoV infection and potential target genes for controlling the infection using zebrafish as model organisms.

In contrast, a group of researchers reported that zebrafish embryos were more efficient and robust than zebrafish larvae in supporting the replication of HuNoV [40]. When injected with the virus, zebrafish embryos showed higher virus levels and continuous replication compared to zebrafish larvae. The use of zebrafish embryos also facilitated easier and quicker manipulation during injection. The replication of HuNoV in zebrafish embryos was followed over time, and the highest virus levels were observed at 2 days post-infection (dpi). Zebrafish larvae, on the other hand, showed a gradual decrease in virus levels after 2 dpi. The zebrafish embryo model was also used to study different HuNoV strains, and similar replication patterns were observed. The zebrafish embryos allowed for continuous passaging of the virus, enabling the study of adaptive mutations. No visual pathological changes were observed in either zebrafish larvae or embryos during virus replication. The zebrafish embryo tool was also effective in studying the binding of HuNoV to human histo-blood group antigens (HBGAs) and evaluating virus inactivation. Compared to stool samples, HuNoVs produced by zebrafish embryos showed clearer binding patterns to HBGAs.

Additionally, Tan et al. (2023) also showed the zebrafish embryo model allowed for the evaluation of virus inactivation, with up to a 2-log reduction achieved using UV irradiation. These findings highlight the potential of zebrafish embryos as a valuable tool for studying HuNoV replication, binding, and inactivation. Furthermore, Cuvry et al. (2022) demonstrated that Lewis X, a fucose-containing histo-blood group antigen (HBGAs), plays an essential role in HuNoV infection in zebrafish larvae by serving as an attachment factor for viral entry [41]. Consistently, inhibition of fucosyl-transferases, resulted in reduced expression of Lewis X, leading to a significant decrease in viral replication. However, contrary to observations in mice and humans, Cuvry et al. (2022) found that the zebrafish microbiota did not enhance HuNoV replication in germ-free larvae.

These studies collectively demonstrate the utility of zebrafish models for studying HuNoV replication and pathogenesis. The larval zebrafish model offers a simpler and more accessible alternative to existing models, providing insights into viral replication, host responses, and potential therapeutic targets. Additionally, zebrafish embryos exhibit robust and continuous HuNoV replication, allowing for the study of different strains, adaptive mutations, binding mechanisms, and virus inactivation methods. These advancements in zebrafish-based research contribute to a better understanding of HuNoV infection and facilitate the development of preventive and therapeutic strategies to combat this significant public health concern.

### 2.6 Herpes simplex virus – type 1 (HSV-1)

HSV-1, or Herpes simplex virus type 1, belongs to the Alphaherpesviridae subfamily and possesses a double-stranded DNA genome [95]. HSV-1 is predominantly transmitted through saliva or other bodily secretions in humans. While commonly associated with cold sores, HSV-1 can also give rise to various herpetic lesions, such as herpetic sycosis, herpes gladiatorum, and herpetic whitlow [96].

To gain deeper insights into HSV-1 infection, researchers have successfully developed a zebrafish model to study the effects of this viral pathogen [42]. This study introduces zebrafish as a novel model for studying herpes simplex virus type 1 (HSV-1) infection and its impact on the nervous system. Burgos et al. (2008) successfully infected adult zebrafish with HSV-1 through intraperitoneal injection. Initially, the infection was localized to the abdominal cavity but later spread to the nervous system, including the brain. Histological analysis revealed the presence of viral antigens in neural regions, accompanied by histopathological alterations and immune system activation. Hemorrhaging in muscle tissue was also observed, indicating a systemic response to the viral infection. To assess the efficacy of antiviral treatment, acyclovir (ACV), which hinders viral DNA synthesis by blocking the viral DNA polymerase, was administered. The results showed that ACV treatment significantly reduced the percentage of infected regions and viral DNA concentrations in zebrafish. However, this study lacks examination regarding whether the administration of ACv influences the immune response or triggers muscle hemorrhages in zebrafish [42].

In the context of HSV-1 infection, the cellular receptor known as human heparan sulfate modifying enzyme 3-*O*-sulfotransferase-3 (3-OST-3) has been identified [97]. Interestingly, zebrafish exhibit the expression of multiple isoforms of 3-OST [43]. Elucidating the functional characteristics of these zebrafish 3-OST isoforms, studies have employed heterologous expression in CHO–K1 (Chinese hamster ovary) cells, which are typically resistant to HSV-1 infection. Remarkably, the introduction of zebrafish 3-OST isoforms into these cells rendered them susceptible to HSV-1 infection [43,29,98,99]. Notably, zebrafish 3-OST isoforms exhibit wide distribution within the central nervous system, indicating the potential utility of zebrafish as an advantageous model for studying the impact of HSV-1 infection on the central nervous system and for evaluating potential therapeutic interventions [29].

The zebrafish model has also been leveraged to examine the dynamics of HSV-1 infection across different stages of larval development, spanning from 48 to 96 h post-fertilization (hpf) [44]. Ge et al. (2015) established an in vivo model using zebrafish to study the cytosolic DNA sensing pathway in response to HSV-1 infection. The study shows that the infection pattern in zebrafish closely resembled that in mice, both in terms of the time course and location of colonization. The authors were also able to optimize the infection time window in zebrafish larvae, reducing the viral dose required for future screening experiments. HSV-1 infection in zebrafish triggered the expression of antiviral genes, indicating the fish's ability to sense the virus and initiate innate immune responses. The RIG-I/MAVS (Retinoic acid-Inducible Gene I/Mitochondrial Antiviral-Signaling protein) signaling pathway, known to respond to RNA viruses, did not affect the antiviral responses to HSV-1 infection, similar to observations in mice. Ge et al. (2015) also found that zebrafish STING (stimulator of interferon genes) played a crucial role in the induction of antiviral genes in response to HSV-1. Silencing zebrafish STING abolished the expression of antiviral genes, which could be rescued by introducing human or mouse STING. Therefore, this study provides a valuable tool to explore the DNA sensing pathway and investigate the conservation and diversification of this pathway across species. It also opens avenues for screening compounds targeting the STING signaling pathway.

As listed above, the zebrafish model has proven to be a valuable tool for studying HSV-1 infection and its effects on the nervous system. It allows for the investigation of viral spread, immune responses, and the evaluation of potential antiviral therapies. The zebrafish model also contributes to our understanding of the cellular receptors involved in HSV-1 infection and the activation of innate immune responses. Overall, these studies demonstrate the usefulness of the zebrafish model in HSV-1 research and highlight its potential for further advancements in the field.

### 2.7 Hepatitis C viruses (HCV)

Hepatitis C, caused by the RNA virus Hepatitis C virus (HCV) from the Flaviviridae family, is an infectious disease that can result in acute or chronic infection [100]. Acute HCV infection can progress to chronic hepatitis C in 50–80 % of cases. Chronic HCV infection initiates a persistent inflammatory process in the liver, which can ultimately lead to liver fibrosis, cirrhosis, hepatocellular carcinoma, and mortality [101]. Notably, Hepatitis C is a primary reason for liver transplantation in various regions worldwide [102,103].

Ding et al. (2011) conducted one of the initial studies utilizing zebrafish as a model organism to examine the replication of HCV and assess the effectiveness of *anti*-HCV drugs. The model involved the co-injection of two vectors containing HCV genes and fluorescent markers into zebrafish zygotes. The successful amplification of the HCV sub-replicon was confirmed by the detection of fluorescence signals in the liver of the larvae. The replication of the sub-replicon was able to be validated through the detection of HCV core mRNA and protein. The sub-replicon exhibited a liver-selective tendency in zebrafish larvae. The zebrafish model also demonstrated transcriptional changes in host genes such as Hsp70, Argsyn, Leugpcr, Rasgbd, chemokine-1, and ScarF2 genes, similar to those observed in HCV-infected human liver cells. The study suggests that zebrafish liver cells provide a supportive environment for HCV replication, making the zebrafish model a promising tool for studying HCV and evaluating potential antiviral drugs.

Building upon these findings, Zhao et al. (2013) introduced a zebrafish model with a construct that co-expresses green fluorescent protein (GFP) and HCV-core genes under liver-specific promoters and enhancers. The model effectively showcases liver-specific expression of HCV core and GFP, confirmed through a range of molecular analyses such as in situ hybridization, RT-PCR, and Western blotting. Importantly, the model also exhibits elevated expression of genes associated with liver pathology related to HCV infection, confirming the functionality of the system.

Recent advancements in the zebrafish model have allowed for the development of a sub-genomic replication system specifically tailored for HCV studies [47]. In this study, they successfully demonstrated the replication of HCV sub-genomic RNA in zebrafish larvae, as indicated by the detection of HCV-encoded proteins and the presence of negative-strand RNA. The observed changes in gene transcription and liver pathology markers, including leptin receptor and heparanase, further confirmed the fidelity of the zebrafish model to HCV infection in human liver cells. Therefore, Ding et al. (2015) proposed that, compared to mouse models, the zebrafish model offers advantages such as active and stable replication, straightforward creation of sub-replicon-positive larvae, and suitability for drug screening. In addition, the researchers also observed the activation of EIF2AK3 (eukaryotic translation initiation factor 2-alpha kinase 3) and ATF6 (activating transcription factor 6) UPR (unfolded protein response) pathway and endoplasmic reticulum (ER) stress response following HCV infection [104]. They identified genes associated with liver pathology that were upregulated in the HCV model larvae, including *ScarF2*, *argsyn*, *rasgbd*, and *LGR5*, indicating the presence of HCV-related pathological features. Furthermore, the study found that different isoforms of the ATG10 (Autophagy-related protein 10) protein had opposing effects on HCV replication, suggesting that targeting ATG10 could be a potential strategy for developing antiviral compounds to complement direct-acting antiviral (DAA) drugs.

Collectively, the zebrafish model has emerged as a valuable platform for studying HCV replication and evaluating potential antiviral drugs. Through the co-injection and expression of HCV genes in zebrafish larvae, researchers have successfully replicated HCV sub-replicons and observed liver-specific expression of HCV-related proteins. These models have provided insights into HCV replication dynamics, transcriptional changes in host genes, and liver pathology associated with HCV infection. The zebrafish model offers advantages over traditional mouse models, making it a promising tool for future studies and drug discovery efforts targeting HCV.

### 2.8 Influenza a virus (IAV)

Influenza viruses, belonging to the Orthomyxoviridae family, are a significant cause of respiratory infections globally. They are categorized into four types (A, B, C, and D) based on distinct internal proteins. Type A and B viruses can cause severe disease in humans, while type C infections are usually mild. Type D viruses primarily infect and reside in cattle, but evidence of infection in humans and other mammals is emerging [105,106]. Type A influenza virus (IAV) is the main cause of epidemics and pandemics in humans and can infect various animal species [107]. IAV is an enveloped virus with eight segments of single-stranded RNA encoding multiple viral proteins. In humans, seasonal IAV primarily infects the respiratory tract, particularly airway epithelial cells, leading to disease dissemination [108]. Although airway macrophages can also be infected, replication of seasonal IAV in these cells is usually limited [109].

Recent studies have highlighted the successful development of zebrafish models that closely mimic the characteristics of human disease during Influenza A virus (IAV) infection [48,25]. In the study reported by Gabor et al. (2014), zebrafish embryos were infected with IAV, leading to increased viral load and mortality compared to uninfected controls. Interferon (IFN) antiviral signaling was induced in infected zebrafish, as evidenced by cytokine profiles and upregulated expression of *ifnϕ1* and *mxa* innate antiviral genes. Microscopic imaging for gross pathology and Toluidine Blue staining for histopathological analysis confirmed typical influenza symptoms, such as edema and tissue damage. Additionally, a GFP reporter strain of IAV allowed visualization of infected cells in vivo, and treatment with the neuraminidase inhibitor Zanamivir reduced GFP expression and mortality, highlighting the model's relevance for drug screening. Therefore, this zebrafish embryo model holds promise for studying host-pathogen interactions and identifying novel antiviral therapies.

Furthermore, Goody et al. (2017) reported that human influenza A virus (IAV) can infect zebrafish muscle fibers, leading to damage characterized by loss of sarcolemma integrity and disruption of extracellular matrix adhesion. They also observed the presence of inflammation markers in muscle tissue following IAV infection [49]. In this study, zebrafish were inoculated with a fluorescent reporter strain of IAV carrying the non-structural protein 1 NS1:GFP fusion protein. Subsequent observation revealed punctate GFP fluorescence distributed across the embryos, thereby showcasing the feasibility of visualizing and tracking infected cells expressing fluorescent products in real-time. This model offers opportunities to further investigate the effects of different factors on IAV tropism and the subcellular localization of viral proteins. Additionally, these findings by Goody et al. (2017) may shed light on the mechanisms and potential treatments for IAV-induced myocarditis, a complication that is challenging to study in mammalian models.

Overall, the utilization of zebrafish models has significantly contributed to our understanding of IAV infection. These models closely mimic human disease characteristics and provide a valuable tool for studying host-pathogen interactions, identifying novel antiviral therapies, and investigating the complications associated with IAV infection. The visualization of viral replication, host immune responses, and the evaluation of therapeutic interventions in zebrafish models have advanced our knowledge of influenza virus pathogenesis and may lead to the development of effective strategies to mitigate the impact of influenza on human health.

### 2.9 Zika virus (ZIKV)

The Zika virus (ZIKV) is a member of the Flaviviridae family, characterized by its positive sense, single-stranded RNA genome enclosed in an envelope [110]. ZIKV primarily spreads to humans through *Aedes aegypti* and *Aedes albopictus* [111]. However, other modes of transmission exist, including sexual intercourse, laboratory exposure, blood transfusion, and transmission from mother to fetus [112]. ZIKV is a significant global health concern with no approved vaccines or treatments available and can cause severe complications such as microcephaly, neurological disorders, thrombocytopenia, and testicular damage [111,112]. Therefore, understanding how ZIKV invades and persists in the brain, particularly in neural progenitor cells, is crucial. However, the mechanisms by which the virus travels and spreads to the brain are still unclear.

In a study by Ayala-Nunez et al. (2019), a xenotypic system was developed to investigate the involvement of infected human monocytes in the dissemination of ZIKV to neural cells using zebrafish embryos modified to express a fluorescent protein in endothelial cells. Human primary monocytes infected with ZIKV were injected into these embryos, and their behavior was observed. The location of transmigrated monocytes did not significantly differ between conditions, but the percentage of extravasated cells was higher when monocytes were exposed to ZIKV [50]. They also analyzed the hemodynamic behavior of circulating monocytes and found that prior exposure to ZIKV increased monocyte track duration and reduced speed. ZIKV-exposed monocytes also exhibited a higher percentage of immobilization onto endothelial surfaces. The use of zebrafish embryos as an in vivo model in this study provided valuable insights into monocyte transmigration that would be challenging to achieve in complex mammals due to technical limitations.

Furthermore, Maleski et al. (2022) established a zebrafish model to investigate the effects of ZIKV infection on embryonic development [51]. The infected larvae exhibited an antiviral immune response, including the production of type I interferons (IFNs). ZIKV infection also induced the production of inflammatory mediators involved in neutrophil recruitment, including IL-1β, IL-6, IL-34 and TNF-α. The expression of inducible nitric oxide synthase (iNOS) genes and IFNs suggested their involvement in infection-induced granulopoiesis. The iNOS genes have been demonstrated to function downstream of the transcription factor C/ebpβ in regulating the proliferation of hematopoietic stem and progenitor cells (HSPCs), which stimulate demand-driven granulopoiesis in response to infection [113]. In addition, ZIKV was successfully introduced into zebrafish embryos, leading to abnormal development and impaired neurological and visual functions. The infected embryos showed morphological alterations and changes in locomotion and swimming patterns, indicative of neurological and visual defects. Importantly, Maleski et al. (2022) reported that the eye development of infected embryos was particularly affected, with damage to the retina and lens and enlargement of the inner nuclear layer. Overall, their findings demonstrate that the zebrafish model effectively recapitulates ZIKV-induced retinopathy during early embryonic development, highlighting the impact on visual and locomotor function.

To sum up, the zebrafish model has emerged as a powerful tool for investigating ZIKV infection and its impact on different aspects of development. These studies have shed light on the behavior of infected monocytes and the effects of ZIKV on embryonic development. These findings contribute to our understanding of ZIKV pathogenesis and may aid in the development of interventions to mitigate the detrimental effects of ZIKV infection.

### 2.10 Human cytomegalovirus (HCMV)

Human cytomegalovirus (HCMV) is a widespread pathogen that can cause severe disease in immunocompromised individuals and congenital infections [114]. Congenital CMV can lead to neurodevelopmental disabilities [115]. The role of specific HCMV proteins in pathogenicity, especially in congenital CMV, is poorly understood due to species-specificity and limitations in animal models. A study reported by Cazorla-Vázquez et al. (2019) assessed the suitability of the zebrafish model for studying the patho-regulatory function of HCMV-encoded proteins [63].

To achieve this, zebrafish embryos at the single-cell stage were injected with plasmids encoding pUL97, an HCMV serine/threonine protein kinase [63]. Microscopic analysis showed mosaic expression of the pUL97, indicating the potential expression in zebrafish embryos. The findings suggest that HCMV-encoded proteins can be expressed in zebrafish. Cazorla-Vázquez et al. (2019) also demonstrate that the observed phenotypes induced by pUL97 are dependent on its kinase activity and may interfere with cellular proliferation and other host cell processes. Although the zebrafish model presented by Cazorla-Vázquez et al. (2019) could not directly enable the study of CMV infection, amplification, and release, it can contribute to understanding the function of individual cytomegaloviral proteins within the cellular machinery of the host.

## 3 Exploration of antiviral compounds using zebrafish

The evaluation of natural compounds and herbal formulations for their potential in combating SARS-CoV-2 using zebrafish as a model has gained significant attention (Table 2). In a study by Zizioli et al. (2023), Cabotegravir (CAB), an HIV integrase strand transfer inhibitor (INSTI), was examined for its impact on zebrafish development. The findings indicate that CAB does not impact the survival or gross morphology of zebrafish larvae at concentrations up to 50 μM. However, higher concentrations led to adverse effects such as pericardial edema, swim bladder underdevelopment, decreased heartbeats, growth delay, and reduced hatching rate. Even at low doses (10 and 20 μM), CAB caused decreased locomotion and disrupted neuronal differentiation in zebrafish larvae, suggesting potential effects on the nervous system [116]. Therefore, Zizioli et al. (2023) suggest that CAB may impact neurodevelopment even at subtherapeutic concentrations, highlighting the importance of evaluating its safety during pregnancy.

**Table 2:** Several compounds showing promising antiviral activities tested in zebrafish as the model of study.

No	Plants/compounds/extracts	Key findings	Refs
1	Cabotegravir	● Cabotegravir (an HIV integrase strand transfer inhibitor) exposed to zebrafish larvae at the concentration of up to 50 μM did not affect the survival of the larvae. ● However, several adverse effects were observed, such as pericardial edema, underdevelopment of bladder, decreased heartbeats (at higher concentration) and decreased locomotion and disruption of neuronal differentiation (at lower concentration).	[116]
2	*Polygonum cuspidatum*	● The water and ethanol extracts of *P. cuspidatum* blocked the entry of SARS-CoV-2 (wild-type and Omicron variants) into zebrafish larvae (IC_50_ of 0.015–0.04 mg/mL). ● This activity might be associated with their ability to disturb the interaction between the spike protein and ACE2 receptor. ● Of several compounds tested, it was found that gallic acid showed a promising antiviral activity.	[117]
3	*Tinospora cordifolia*	● Several pathological events observed in zebrafish after being exposed to the spike protein of SARS-CoV-2 experienced improvements (e.g., swim bladder morphology was restored and reduced infiltration of immune cells). ● Improvements of survival rates and behavioral fever were also mitigated after treatment with this herb.	[73]
4	Coronil	● Three extracts from *Withania somnifera*, *Tinospora cordifolia*, and *Ocimum sanctum* were combined and given to zebrafish infected with SARS-CoV-2. ● This product showed promising actions in reducing cytokine production, mitigating behavioral fever, preventing edema and promoting recovery of the damaged renal cells.	[53]
5	*Stenocline ericoides* and *Stenocline inuloides*	● Methanolic extract of these plants showed *anti*-ZIKV activity tested in A549 cells with IC_50_ of 58.1 μg/mL (*S. ericoides*) and 63.5 μg/mL (*S. inuloides*). ● Methanolic extract of these plants showed *anti*-DENV activity tested in Huh7.5 cells with IC_50_ of 36.2 μg/mL (*S. ericoides*) and 49.7 μg/mL (*S. inuloides*). ● Different antiviral mechanisms against ZIKV and DENV were shown by each extract. While *S. ericoides* neutralized the infectivity of those viruses, *S. inuloides* prevented the attachment of the viruses to the host. ● No acute toxicity was observed in zebrafish after being exposed to the extracts (100 μg/g of body weight).	[118]
6	Cranberry pomace	● Cranberry pomace extract demonstrated antiviral activities against DENV-2 (IC_50_ 54.2 μg/mL), DENV-3 (IC_50_ 40.3 μg/mL) and ZIKV (IC_50_ 26 μg/mL) tested in human Huh7.5 and A549 cell lines, respectively. ● *Anti*-ZIKV activity of the extract was related to the binding inhibition of the virus to the host cells. ● After being tested in adult zebrafish, the extract of cranberry pomace (400 μg/g of body weight) did not show toxic effects.	[119]
7	*Phyllantus phillyreifolius*	● Tested in vitro in A549 cells, ethanolic extract of *P. phillyreifolius* showed antiviral activity against ZIKV (IC_50_ = 55 μg/mL), while the major component of the extract, geraniin, showed *anti*-ZIKV activity with IC_50_ 22 μg/mL. ● Tested in vitro in human hepatoma cells, Huh7.5, the extract of P. phillyreifolius (250 μg/mL) and geraniin (200 μg/mL) showed *anti*-DENV activity. ● Zebrafish was used to study in vivo acute toxicity of the extract (375 μg/g of body weight) and geraniin (300 μg/g of body weight) showing no acute effects generated after exposure. ● The putative antiviral mechanism was exerted by blocking the penetration of the viruses into the hosts.	[120]
8	*Ayapana triplinervis*	● Studied in A549 cells, essential oil (IC_50_ = 38 μg/mL) of *A. triplinervis* and its main compound namely THQ (IC_50_ = 45 μg/mL) were linked to antiviral activity against ZIKV. ● At its antiviral effective concentration (150 μg/g of body weight), THQ was exposed to zebrafish. No acute toxicity was observed, while the viability of the fish was not affected as well.	[120]
9	*Fucus vesiculosus*	● Fucoidan extracted from *F. vesiculosus* exhibited antiviral activities agains HuNoV infected to zebrafish. ● It has been shown that the fucoidan inhibited replication of the virus in zebrafish larvae. ● This action could be linked to the ability of the fucoidan to enhance the innate immune response through upregulation of genes encoding antiviral mediators such as *ilf2* and factors regulating antiviral innate immune response such as *vamp8, syk, sting1*, *mvp,* and *unc93b1*.	[121]

Abbreviations: ACE2, Angiotensin-converting enzyme 2; DENV, Dengue virus; HIV, human immunodeficiency virus; HuNoV, Human Norovirus; IC50, Half-maximal inhibitory concentration; SARS-CoV-2, Severe Acute Respiratory Syndrome Coronavirus 2; THQ, thymohydroquinone dimethyl ether, ZIKV, Zika virus.

Lin et al. (2022) found that extracts of *Polygonum cuspidatum* exhibited potent antiviral effects against wild-type and omicron variants of SARS-CoV-2. The extracts were effective in blocking multiple steps of virus entry, including the interaction between the spike protein and the ACE2 receptor, as well as the activity of the 3CL (3-chymotrypsin-like) protease. Among the tested compounds, gallic acid, found in *P. cuspidatum*, demonstrated significant antiviral activity, suggesting its contribution to the overall efficacy of the herbal extract. Based on these findings, *P. cuspidatum* extracts have the potential to be considered as promising treatments for COVID-19 [117].

In investigating the therapeutic potential of *Tinospora cordifolia*, Balkrishna, Khandrika et al. (2021) found that this herb could alleviate certain pathological effects induced by the SARS-CoV-2 spike protein in zebrafish. *T. cordifolia* treatment restored the swim bladder morphology, reduced immune cell infiltration, and reversed skin hemorrhage. However, its effectiveness in rescuing kidney damage was limited. *T. cordifolia* also mitigated behavioral fever and improved survival rates, showing dose-dependent effects. In conclusion, *T. cordifolia* showed promise in reversing certain pathological effects induced by the spike protein, although its efficacy varied across different organs and tissues [73].

Similarly, Balkrishna et al. (2020a) studied the therapeutic potential of Coronil, a tri-herbal medicine composed of extracts from *Withania somnifera*, *Tinospora cordifolia*, and *Ocimum sanctum*, in treating SARS-CoV-2-induced pathologies. The study utilized in silico, in vitro, and in vivo approaches to assess the immunomodulatory, anti-inflammatory, and antiviral properties of Coronil. Results demonstrated that Coronil reduced cytokine production, attenuated behavioral fever, prevented edema, and promoted recovery from renal cell damage in a zebrafish model. These effects were attributed to the anti-inflammatory and immunomodulatory properties of the herbal components present in Coronil [53].

Employing comparable methodologies and model system, the researchers also documented the effectiveness of the concentrated extract derived from *Withania somnifera* and Divya-Swasari-Vati (DSV), a calcium-rich herbal mixture prescribed in Ayurvedic medicine for respiratory conditions, in mitigating pathological responses induced by the recombinant spike (S) protein of SARS-CoV-2 in a humanized zebrafish model [53,54]. These studies underscore the potential of natural compounds and herbal formulations in combating SARS-CoV-2 infection and associated pathologies. The utilization of zebrafish as a model organism has proven to be valuable in screening the safety and efficacy of these interventions. The zebrafish model offers a cost-effective and efficient platform for studying the effects of various compounds on SARS-CoV-2 pathogenesis, allowing for rapid screening of potential treatments. Continued exploration of zebrafish models may lead to the discovery of novel therapeutic candidates and contribute to the ongoing efforts to mitigate the global impact of SARS-CoV-2.

In recent years, a multitude of studies have concentrated on investigating the antiviral properties of plant extracts or compounds in vitro, followed by assessing their in vivo toxicity using zebrafish as a reliable model organism. In the pursuit of natural antiviral agents against ZIKV and DENV infections, several reports have documented the potential of various plant extracts and compounds.

Endemic to Madagascar, *Stenocline ericoides* and *Stenocline inuloides*, two plant species with rich biodiversity, were subjected to investigation. Phytochemical analysis revealed that both species are abundant in polyphenols and flavonoids, hinting at their potential as sources of antiviral compounds [118]. Upon further examination, it was discovered that the extracts of *S. ericoides* and *S. inuloides* exhibited distinct mechanisms of action against ZIKV and DENV. The former extract effectively neutralized the infectivity of both viruses, while the latter extract demonstrated a preventive effect on viral binding in human A549 cells. Remarkably, no acute toxicity was observed in zebrafish assays, suggesting the safety of these extracts at effective antiviral concentrations. The results suggest that Stenocline extracts hold promise as natural remedies against medically significant flaviviruses.

In parallel, cranberry pomace (CP) extract was investigated for its antiviral properties [119]. The extract not only displayed antiviral activity against ZIKV in human A549 cells but also inhibited viral binding and exhibited virucidal effects. Encouragingly, no signs of suffering, stress, or abnormal behavior were observed when the CP extract was intraperitoneally injected into zebrafish at the maximum non-toxic concentration. These findings further establish CP extract as a potential natural antiviral agent against medically relevant flaviviruses.

Similarly, the ethanolic extract of *Phyllanthus phillyreifolius* and its major compound, geraniin, showed great promise as potent antiviral agents against ZIKV and DENV [120]. The extract and geraniin effectively inhibited ZIKV infection in human cell lines in a concentration-dependent manner, with complete inhibition at non-cytotoxic concentrations. Furthermore, they displayed potent antiviral activity against all four serotypes of DENV. Interestingly, the extract and geraniin primarily targeted the early stages of ZIKV infection, particularly virus entry into host cells. Additionally, zebrafish toxicity tests confirmed the absence of acute toxicity at the maximum non-toxic concentrations tested, further reinforcing their potential as natural antiviral agents.

Expanding the scope, *Ayapana triplinervis* essential oil (EO) and its main compound, thymohydroquinone dimethyl ether (THQ), exhibited significant antiviral activity against ZIKV [120]. Non-cytotoxic doses of *A. triplinervis* EO effectively inhibited ZIKV infection in human lung epithelial cells, while THQ demonstrated potent inhibition of viral infection as well. Mechanistically, THQ acted at the early stages of ZIKV infection, specifically preventing viral entry and internalization into host cells. Encouragingly, in vivo experiments using zebrafish showed no toxic effects when administered the antiviral dose of THQ. In summary, these studies collectively highlight the potential of *Stenocline* extracts, cranberry pomace extract, *P. phillyreifolius* extract (particularly geraniin), and *A. triplinervis* essential oil (specifically THQ) as natural antiviral agents against ZIKV and DENV. These natural compounds demonstrate significant inhibitory effects on viral infection and hold promise as potential therapeutic interventions.

The exploration of functional food components as natural therapeutic agents has gained attention in the search for effective treatments. Fucoidan is a type of polysaccharide derived from brown seaweeds, containing significant amounts of fucose and sulfate ester groups [122]. A series of studies investigated the effects of fucoidan from *Fucus vesiculosus* on HuNoV infection in zebrafish larvae [123,121]. The results showed that fucoidan inhibited HuNoV replication in zebrafish larvae and induced the upregulation of innate immune response genes through upregulation of genes encoding antiviral mediators such as *ilf2* and factors regulating antiviral innate immune response such as *vamp8, syk, sting1, mvp*, and *unc93b1*. Pathway analysis also revealed enrichment of the innate immune system gene cluster. Overall, the findings suggest that fucoidan enhances the host's innate immune response and mitigates HuNoV infection [123,121]. Therefore, this research opens up new avenues for the development of natural antiviral interventions by harnessing the power of functional food components. Fucoidan, with its unique properties and natural origin, holds promise as a potential therapeutic agent against enteric virus infections, providing a novel approach to bolstering the innate immune system's ability to combat viral pathogens.

## 4 Limitations of zebrafish as a model for human viral infections

**Fig. 3:**
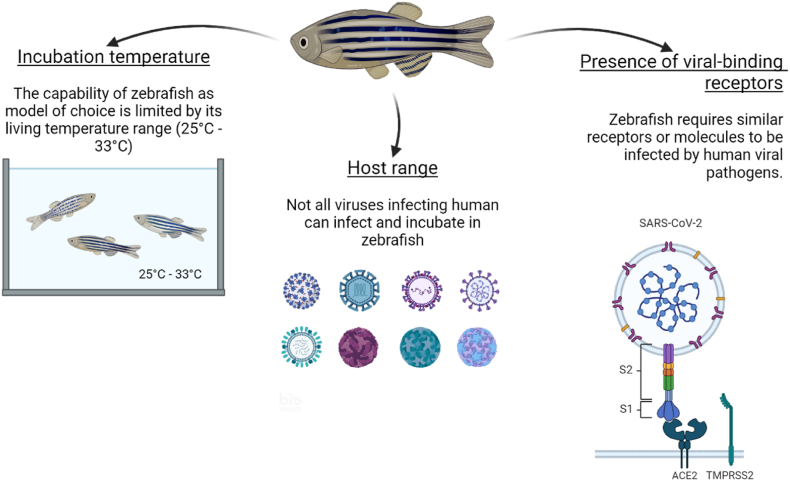
Challenges in utilizing zebrafish as a model for human viral infections. Zebrafish models are valuable for studying human viral infections, but their evolutionary differences from humans limit the range of viruses that can be effectively investigated. Key factors to consider include the viral incubation temperature, host range, and presence of zebrafish orthologs of human viral receptors. Human viruses thrive at 37 °C, while zebrafish live at 25–33 °C, making them suitable hosts only for viruses that replicate well within this temperature range. Additionally, viruses with a broad host range are more likely to infect zebrafish, and the presence of conserved viral receptors in zebrafish is essential for effective infection.

Zebrafish models have emerged as valuable tools for investigating human viral infections [21]. However, due to the evolutionary divergence between zebrafish and humans, the scope of viral infections that can be effectively studied in this model system is limited (Fig. 3). To determine the suitability of zebrafish as an infection model for specific human viral pathogens, crucial considerations include viral incubation temperature, host range, and the presence of zebrafish orthologs of known viral receptors [22]. Human viral pathogens are adapted to thrive at the warmer body temperature of humans at 37 °C. Meanwhile, zebrafish can tolerate a range of temperatures between 25 and 33 °C [124]. Consequently, zebrafish may only serve as viable host candidates for a subset of viruses capable of robust replication within their temperature range. Moreover, host range is a crucial determinant in the feasibility of infecting zebrafish with viral pathogens. Viruses with a broad host range exhibit a higher likelihood of successfully infecting zebrafish. Conservation of viral receptors is also paramount, as these molecules mediate viral entry and subsequent infection. Zebrafish must possess equivalent receptors or orthologous molecules to facilitate infection by human viral pathogens. Considering these limitations enables targeted selection of viruses for infection studies, enabling in-depth exploration of virus-host interactions and evaluation of potential antiviral interventions within the zebrafish model system.

## 5 Conclusion and future perspectives

In conclusion, zebrafish models have emerged as valuable tools for studying viral pathogenesis and understanding the complex interactions between viral pathogens and their hosts. The use of zebrafish as a research model offers several advantages, including a well-characterized immune system that shares similarities with humans, the ability to visualize infected cells and immune responses in real-time, and the opportunity to study viral infection dynamics and host responses [20,21,23]. Studies utilizing zebrafish models have provided significant insights into the pathogenesis of various viral infections as reviewed here. For example, investigations using zebrafish models have shed light on the dynamics of viral spread, immune responses, and organ-specific manifestations of infections caused by viruses such as CHIKV, SINV, DENV, HuNoV, HSV-1, HCV, and IAV. The zebrafish models have offered significant insights into viral pathogen replication, their impact on host tissues and organs, the activation of immune responses, and the assessment of potential therapeutic interventions. These findings have enhanced our understanding of viral pathogenesis, identified novel targets for drug development, and provided valuable platforms for testing antiviral drugs.

As research in the field of viral pathogenesis continues to advance, zebrafish models are expected to play an increasingly prominent role. Future research directions can be focused on various aspects of utilizing zebrafish as a model to study viral infections. Firstly, improving the zebrafish model's effectiveness in studying viral pathogenesis by exploring its immune system and similarities to humans. This includes investigating immune cell types, conserved immune pathways, and the zebrafish's response to viral infections. Secondly, conducting comparative analyses of viral pathogenesis using zebrafish models to understand similarities and differences among different viruses. Such investigations would entail scrutinizing the similarities and distinctions in viral pathogenesis across different viruses, including but not limited to SARS-CoV-2, CHIKV, SINV, DENV, HuNoV, HSV-1, HCV, and IAV. This can provide valuable insights into viral replication, host responses, and disease outcomes. Thirdly, unraveling host-virus interactions and immune responses during viral infections in zebrafish to identify potential therapeutic targets and strategies to modulate immune responses for better viral control. Fourthly, investigating co-infections and their impact on viral pathogenesis and disease outcomes to inform the development of broad-spectrum antiviral and antibacterial therapies. Lastly, evaluating antiviral therapies and drug development using zebrafish models to discover new antiviral agents and develop more effective treatment strategies. These research directions collectively contribute to advancing our knowledge of viral pathogenesis and host immune responses, leading to improved strategies for combating infectious diseases.

## CRediT authorship contribution statement

**Sofyantoro Fajar:** Writing – review & editing, Writing – original draft, Visualization, Validation, Investigation, Formal analysis, Data curation, Conceptualization. **Sendi Priyono Dwi:** Writing – review & editing, Writing – original draft, Validation, Methodology, Investigation, Formal analysis. **Indah Septriani Nur:** Writing – review & editing, Writing – original draft, Validation, Investigation. **Aristyaning Putri Wahyu:** Writing – review & editing, Writing – original draft, Investigation, Data curation. **Mamada Sukamto S:** Writing – review & editing, Writing – original draft, Visualization, Validation, Investigation. **Adipuri Ramadaningrum Winda:** Writing – review & editing, Writing – original draft, Investigation, Formal analysis. **Wijayanti Nastiti:** Writing – review & editing, Writing – original draft, Supervision, Formal analysis, Conceptualization. **Frediansyah Andri:** Writing – review & editing, Writing – original draft, Supervision, Formal analysis, Conceptualization. **Nainu Firzan:** Writing – review & editing, Writing – original draft, Supervision, Funding acquisition, Conceptualization.

## Declaration of competing interest

The authors declare that they have no known competing financial interests or personal relationships that could have appeared to influence the work reported in this paper.
